# Protection Against Invasive Infections in Children Caused by Encapsulated Bacteria

**DOI:** 10.3389/fimmu.2018.02674

**Published:** 2018-11-20

**Authors:** Manish Sadarangani

**Affiliations:** ^1^Vaccine Evaluation Center, BC Children's Hospital Research Institute, Vancouver, BC, Canada; ^2^Division of Infectious Diseases, Department of Pediatrics, University of British Columbia, Vancouver, BC, Canada

**Keywords:** sepsis, bacteremia, meningitis, *Neisseria meningitidis*, *Haemophilus influenzae*, *Streptococcus pneumoniae*, Group B Streptococcus, *Streptococcus agalactiae*

## Abstract

The encapsulated bacteria *Streptococcus pneumoniae, Neisseria meningitis, Haemophilus influenzae*, and *Streptococcus agalactiae* (Group B Streptococcus) have been responsible for the majority of severe infections in children for decades, specifically bacteremia and meningitis. Isolates which cause invasive disease are usually surrounded by a polysaccharide capsule, which is a major virulence factor and the key antigen in protective protein-polysaccharide conjugate vaccines. Protection against these bacteria is largely mediated via polysaccharide-specific antibody and complement, although the contribution of these and other components, and the precise mechanisms, vary between species and include opsonophagocytosis and complement-dependent bacteriolysis. Further studies are required to more precisely elucidate mechanisms of protection against non-type b *H. influenzae* and Group B Streptococcus.

## Introduction

Encapsulated bacteria have been responsible for the majority of bacteremia and meningitis in children for many decades (1–3). In children aged >3 months, *Streptococcus pneumoniae* (pneumococcus), *Neisseria meningitidis* (meningococcus), and *Haemophilus influenzae* have been the predominant pathogens, with *H. influenzae* being a less significant problem since the introduction of vaccines against type b organisms. Disease is most common in young children <5 years of age and older adults aged >65 years. In neonates and young infants, Group B Streptococcus (GBS) is the major cause of bacteremia and meningitis (1). These organisms have the shared characteristic of being surrounded by a polysaccharide capsule, which is a key virulence factor because it helps the bacteria evade complement deposition and subsequent phagocytosis and killing. These polysaccharides have also been the basis for successful vaccines against all except GBS, because immune responses against the polysaccharide capsule are the primary mechanism of protection for the human host. Each species can be encapsulated by polysaccharides of different biochemical compositions, which has been used for categorization into capsular groups or serotypes.

**Figure 1:**
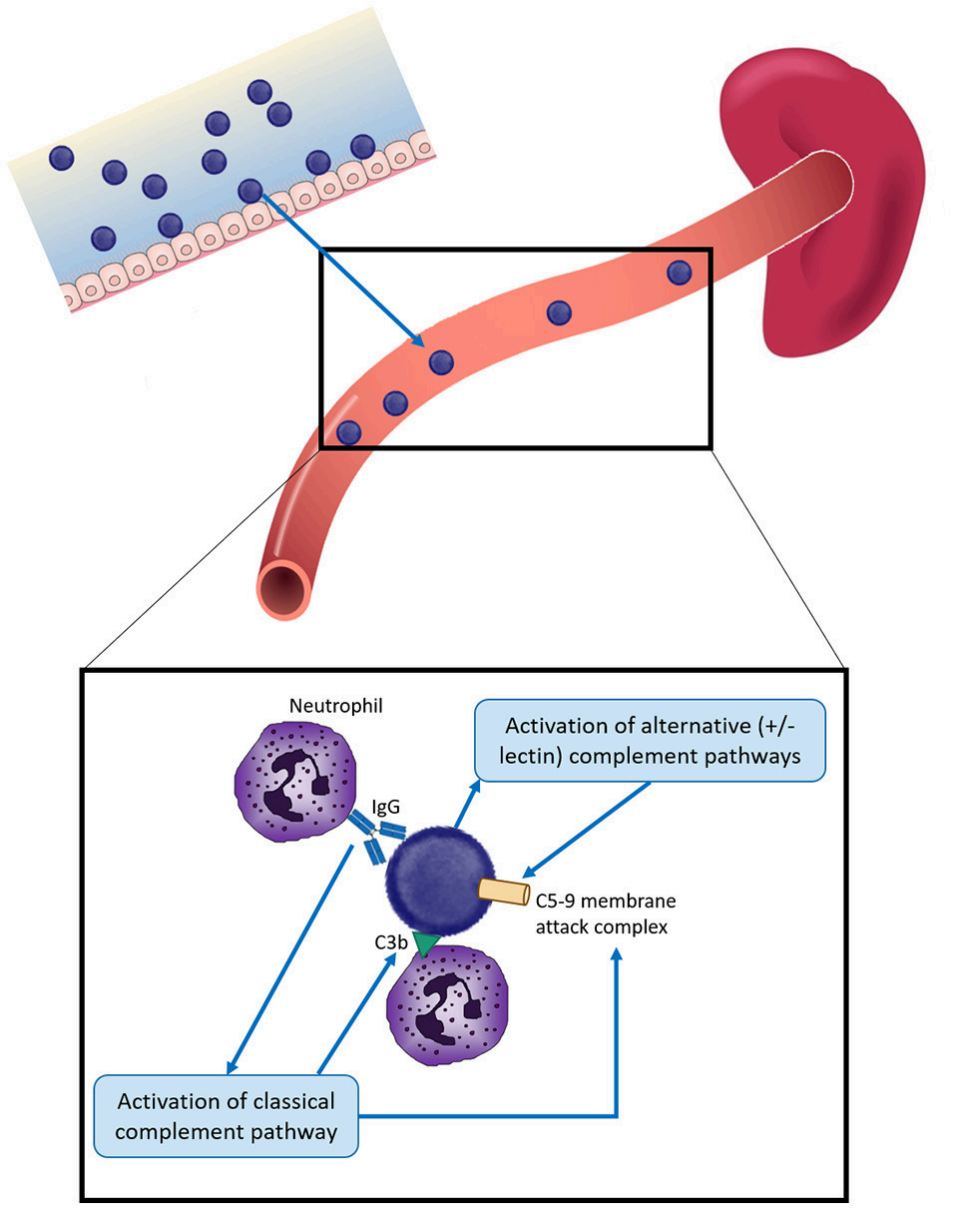
Overview of protection against infection by encapsulated bacteria in children. Encapsulated bacteria initially infect the mucosal surface—nasopharynx for *S. pneumoniae, N. meningitidis*, and *H. influenzae* and the gastrointestinal or vaginal tracts for Group B Streptococcus (GBS). Occasionally bacteria enter the bloodstream to cause severe infection. Protection against invasive infection includes: direct activation of the alternative and/or lectin complement pathways, resulting in insertion of the C5-9 membrane attack complex into the bacterial membrane and bacterial death; binding by specific antibody and activation of the classical complement pathway, resulting in bacteriolysis by the C5-9 membrane attack complex and/or complement C3b deposition. Both antibody and C3b can result in opsonophagocytosis, most commonly by neutrophils. The spleen plays a key role by facilitating phagocytosis and producing components of the complement cascade. Different mechanisms have different relative importance depending on the pathogen, as described in the text.

Protection against these organisms is highly dependent on circulating serum antibody because of the rapid development of disease following infection, which can result in death within hours (Figure 1). While vaccination and/or infection with these organisms does result in development of B cell memory, at least 2–7 days is required following pathogen exposure for a detectable response to occur—which is too slow to mediate protection (4–7). B cell immunity is important to protection against encapsulated bacteria, with common themes across responses to all of the polysaccharide capsules discussed further below. The majority of B cell responses are T-dependent, but responses to polysaccharides are T-independent. Cross-linking of the surface-expressed B cell receptor results in differentiation of polysaccharide-specific B cells into plasma cells, without generation of memory B cells and thus depletes the naïve B cell pool from which future memory cells must be derived (8, 9). As a result, polysaccharide vaccines are generally poorly immunogenic in young children (under 2 years of age), there is no memory generated and no anamnestic response on future exposure to pathogen or booster vaccine doses (10–12). The development of conjugation chemistry, whereby polysaccharide antigens are linked to carrier proteins, resulted in vaccine antigens which recruited T cell help and thus generation of polysaccharide-specific memory B cells even in young children, and which can provide rapid responses upon administration of future vaccine doses (9). Complement and the spleen also play a critical role in protection against encapsulated bacteria. The spleen has a central role in protection against infection by encapsulated bacteria, via phagocytosis and production of opsonins and components of the complement pathway (Figure 1). Asplenic or hyposplenic individuals (e.g., post-splenectomy, sickle cell disease) are therefore rendered at much higher risk of life-threatening infection, reflected by the increased rates of infection by *S. pneumoniae* in particular—but also other encapsulated bacteria such as *H. influenzae* and *N. meningitidis* (13–19). Such individuals are therefore recommended to receive vaccination against all of these three pathogens, even when not in a high risk age group, in addition to long-term antibiotic prophylaxis to prevent infection (19).

## Streptococcus pneumoniae

### The role of antibody, including opsonophagocytosis

The uptake and killing of pneumococci by phagocytic cells, opsonophagocytosis (OP), is thought to be the predominant mechanism of bacterial killing. OP can be mediated by antigen-specific antibody or complement bound to the bacterial surface. OP against pneumococci is largely mediated via neutrophils (Figure 1). Following opsonization by antibody or complement component C3b on the bacterial surface, bacteria are phagocytosed by neutrophils and killed via serine proteases contained within neutrophil granules (20). Antibody coats the bacterial surface, following which the Fc portion binds to neutrophil receptors, initiating bacterial uptake.

In clinical trials of pneumococcal conjugate vaccines (PCVs), the protective antibody threshold was estimated to be 0.35 mg/mL polysaccharide-specific IgG (based on data from 3 clinical trials) and this has been used as the basis for licensure of these vaccines (21–24). A more recent analysis suggested that the actual amount of antibody required for protection varies depending on serotype, between 0.14 and 2.83 mg/mL for the 13 serotypes in the 13-valent PCV (PCV13) (25). In children immunized with the 7-valent PCV (PCV7) at 2, 4, 6, and 12 months of age, antibody levels increased following the 12-month booster, declined slightly by age 24 months and then did not decline much further to age 60 months (26). Antibody levels against serotypes which were frequently seen in carriage (and therefore presumed to be highly circulating) were higher, suggesting that ongoing exposure to *S. pneumoniae* was important in maintaining antibody levels. As well as increasing the total level of antibody, a booster vaccine dose at 12 months of age results in affinity maturation, resulting in higher avidity antibodies. This has been reported with booster doses of both conjugate and polysaccharide vaccines following priming doses with conjugate vaccine in infants (26–28).

The predominant anti-polysaccharide antibody in unvaccinated adults is IgG2 (29). This in part explains the high susceptibility of young children to pneumococcal disease, since IgG2 production in the first 2 years of life is low (30). Although IgG2 provides some immunity against pneumococcal infection it is less efficient than other IgG subclasses in facilitating OP. IgG1 is up to 1,000-fold more efficient in inducing pneumococcal killing compared with IgG2 because IgG1 is a much more effective activator of the complement cascade (31–33). The ability of IgG2 to mediate protection against pneumococci is also impaired by its dependence on a polymorphic Fc receptor, Fcγ-IIA receptor. A single nucleotide polymorphism results in either a histidine (Fcγ-IIA-H131) or arginine (Fcγ-IIA-R131) residue at amino acid 131, with Fcγ-IIA-H131 having a higher affinity for IgG2 compared with Fcγ-IIA-R131 (34, 35). Fcγ-IIA-R131 is present in 30–50% of Caucasian populations (36). Adults who are homozygous for Fcγ-IIA-R131 are more likely to develop severe pneumococcal disease, and this form of the Fc receptor is also more commonly found in those with bacteremic pneumococcal pneumonia compared with healthy individuals and patients with non-bacteremic pneumonia (37–39). Most studies of IgG2-mediated OP have used post-vaccination serum (40, 41), and it is possible that natural immunity also relies on antibody against other bacterial components. In serum from unvaccinated individuals the classical complement pathway has a greater influence on OP than antibody level, and was more reliant on natural IgM, suggesting that IgG may be less important in natural immunity (42). This may also explain why rates of IPD are high in adults aged over 65 years despite high levels of anti-capsular IgG (43).

Natural IgM is encoded by germ-line genes which have not undergone somatic hypermutation. The B-cell repertoire of natural IgM is limited, of low affinity and does not adapt as a consequence of antigen-specific interactions (44). Such antibodies are cross-reactive and recognize antigens common to several pathogens, such as phosphorycholine found in the cell wall of the pneumococcus. Mice depleted natural IgM-producing B cells are highly susceptible to pneumococcal infection (45).

### The role of complement

The importance of complement is highlighted by increased susceptibility to pneumococcal infections in individuals with complement pathway defects (46). In humans, activation of the classical complement pathway predominates in protection against pneumococcal infections, with some contribution from the alternative pathway. This has been confirmed by *in vitro* opsonization studies of pneumococci using serum from patients with complement C2 deficiency or depleted of C1q or factor B (42). Similar data have been obtained in mouse studies (47). The importance of the mannose-binding lectin (MBL) pathway differs between mice and humans. In mice the role of the MBL pathway is thought to be negligible whilst in humans mutations in the *MBL* gene are found in higher frequencies in individuals with IPD in comparison to healthy individuals (48).

*In vitro* anti-polysaccharide antibody alone can facilitate OP for some serotypes, but the addition of complement markedly increases phagocytosis (49, 50). This suggests that antibody-mediated killing occurs through both activation of the complement pathway as well as direct initiation of OP (42). The relative contribution of these two pathways in IgG-mediated protection is not clear. In a mouse model antibody-mediated protection was not reduced when antibody-dependent OP was blocked, whereas complement depletion resulted in bacteremia. These data suggest that IgG-mediated protection is predominantly through complement activation (51). In human sera it has been demonstrated that complement activation may be more important for the protection via IgG1 compared IgG2, in experiments where complement is depleted by heat inactivation (52).

In addition to its role in OP, complement C3b additionally stimulates B cells to increase antibody production via CD21 (53). The complement protein C5b enhances vascular permeability and chemotaxin release that guides neutrophils to the site of infection. The membrane attack complex (consisting of terminal complement components C5-9) is a major endpoint of the classical complement pathway, but does not play a significant role in protection against *S. pneumoniae*, unlike *N. meningitidis* (see below). The reasons for this are unclear, but may be related to the different structure of the Gram-positive *S. pneumoniae* where the cell wall is the predominant outer structure, compared with the Gram-negative *N. meningitidis* where the outer membrane surrounds a smaller cell wall.

### Non-polysaccharide directed immunity

There is some evidence that antibodies against pneumococcal proteins may mediate some protection against pneumococcal infection. Firstly, IgG targeting different pneumococcal proteins, such as pneumolysin, are reduced in older compared with younger adults. Secondly, anti-pneumolysin IgG from patients with pneumonia confer protection against IPD in mice in passive protection studies (54). Finally, the age-related decline in rates of IPD after the age of 2 years is uniform across all serotypes, not only those that are more likely to be found causing carriage, suggesting non-polysaccharide-mediated protection (55).

## Neisseria meningitidis

### The role of antibody, including bacteriolysis

Disease incidence and activity of complement-dependent serum bactericidal antibody (SBA) show an inverse correlation, with the level of SBA being highest at birth and among adults, and lowest in children aged between 6 months and 2 years when the highest incidence of disease occurs (56, 57). Such antibodies occur naturally following asymptomatic carriage of both pathogenic and non-pathogenic Neisseria, such as *Neisseria lactamica*, and other antigenically related Gram negative bacteria. Polysaccharide-protein conjugate vaccines are available for capsular groups A, C, W, and Y; however only protein vaccines are available for group B because the polysialic acid capsule resembles human neuronal cell adhesion molecule and is therefore not immunogenic as a vaccine antigen (58). For the meningococcal capsular group C conjugate vaccine, an SBA titer of ≥8 correlated strongly with postlicensure vaccine effectiveness (59). Following immunization with the capsular group C conjugate vaccine, protection after infant immunization does not persist into the second year of life (60), whereas immunization at age 12 months results in 1–2 years of protection (61), immunization at 1–9 years of age provides 2–5 years of protection, and the most durable responses resulting in ≥5 years of protection occur in children vaccinated when 10 years or older (62–65). For capsular group B disease, the proportions of capsular group B vaccine recipients with ≥4-fold rises in SBA following vaccination or SBA titers ≥4 have been correlated with clinical effectiveness in studies of outer membrane vesicle vaccines (66–68). The predicted effectiveness of a group B protein vaccine in the UK based on SBA (69) was supported by an observational study after vaccine introduction (70), further supporting these thresholds for protection. These cutoffs are, therefore, currently used for regulatory approval of new meningococcal vaccines. Previous studies have reported variable correlation between total antibody and SBA, highlighting the importance of antibody function for protection against meningococcal disease (67, 71–80). Hence, total antibody level is not considered relevant when considering protection against meningococcal disease. The strong association between disease risk and genetic variation in human complement factor H (81), further supports the importance of complement-mediated protection against disease.

### The role of complement

Complement is a key factor in protection against meningococcal disease. The risk of developing meningococcal disease in individuals with primary immunodeficiency who have reduced or absent levels of properdin, factor D, or terminal complement components is up to 1,000-fold higher compared with the rest of the healthy population. Disease risk is also higher in individuals with nephrotic syndrome, systemic lupus erythematosus, hepatic failure, and other diseases which are associated with acquired decreases in levels of complement components, and in patients treated with eculizumab, a monoclonal antibody against complement protein C5. In individuals with complement deficiencies, disease tends to occur during late childhood and adolescence, concordant with higher rates of nasopharyngeal carriage. In addition, infections may be recurrent, which is extremely rare in otherwise healthy individuals. Meningococcal disease cases in those with complement defects are frequently reported as being less severe than in complement-sufficient persons (82), with the exception of properdin deficiency and occasionally in those with late complement component deficiency, perhaps because these cases are often caused by unusual capsular groups. In one study, one-third of individuals with meningococcal disease caused by capsular groups X, Y, and W had a complement deficiency, and group B disease (common in resource-rich countries) has only occasionally been described in series of complement-deficient individuals with IMD. Extensive complement activation and bacteriolysis are protective against early infection, but likely contribute to the pathogenesis of severe disease following bacterial invasion.

### Other mechanisms of protection

There is evidence that mechanisms other than complement-dependent bactericidal antibodies may be important in determining protection against meningococcal disease. Firstly, the relationship between incidence of disease and prevalence of SBA have not been observed in studies in the UK and Canada (83, 84), After the first 2 years of life, disease incidence declined through childhood, but this was surprisingly not associated with an increase in bactericidal antibodies. In the UK study, the second, smaller peak of disease occurring in adolescents and young adults coincided with a paradoxical increase in the proportion of individuals with a protective SBA titer of ≥1:4 and adults had a low risk of disease despite a much lower prevalence of protective bactericidal antibodies. Secondly, in a large study in Iceland SBA titres underestimated vaccine efficacy (85). Thirdly, disease in individuals with complement deficiency has a different age distribution, is often less severe, and often involves unusual capsular groups (82).

Alternative important factors in protection include OP (86) and antibody avidity (77), but there are no data linking these mechanisms with either vaccine efficacy or effectiveness, as has been found with SBA. Protection in the absence of SBA activity is probably conferred by OP (87). This is observed in rats who are deficient in complement factor C6, where opsonization can occur, but bacteriolysis cannot. Both SBA and OP activity occur in animals and humans after immunization with protein and lipopolysaccharide (LPS), but there are few data to suggest that OP without SBA can prevent meningococcal disease or improve outcomes. The importance of antibody avidity and the ability of vaccines to stimulate avidity maturation has been demonstrated for MenC (87). SBA did not correlate well with IgG titres after vaccination with an OMV vaccine, possibly because only high avidity antibodies were bactericidal (88).

The risk ratio for siblings of individuals with invasive disease due to *N. meningitidis* is similar to that for other diseases where susceptibility shows polygenic inheritance. Multiple host genetic factors have been identified which influence either susceptibility to or severity of disease. The molecules implicated involved polymorphisms in genes expressed at epithelial surfaces, the complement cascade, pattern recognition receptors, clotting factors, or inflammatory mediators. Deficiencies in the complement pathways are consistently associated with an increased risk of meningococcal disease, with specific polymorphisms in MBL, and factor H found to be associated with disease susceptibility. A genome-wide association study of 7,522 individuals in Europe identified single-nucleotide polymorphisms within genes encoding complement factor H *(CFH)* and CFH-related protein 3 (*CFHR3*), which were associated with host susceptibility to meningococcal disease (81). Complement-mediated bacteriolysis is known to be extremely important in protection against meningococcal disease, giving these associations biologic plausibility. In particular, factor H attaches to various binding proteins expressed on the bacterial surface, downregulating complement activation and allowing the organism to evade host responses.

## Haemophilus influenzae

### *Haemophilus influenzae* type b

In concurrence with the other encapsulated bacteria, the most important mediator of host defense against *H. influenzae* type b (Hib; the best studied serotype) is antibody directed against the type b capsular polysaccharide polyribosylribitol phosphate (PRP). Anti-PRP antibody is acquired in an age-related fashion and mediates bacterial killing, in part via OP. The importance of anti-polysaccharide antibodies comes from studies where administration of immunoglobulin enriched for these antibodies protected against disease, in both humans and rats (89, 90). Further studies in rats demonstrated that both IgG1 and IgG2 had equivalent functional activities, suggesting multiple effector mechanisms (91). Antibodies against non-polysaccharide antigens, such as OMPs, have also been demonstrated to be protective in the rat model (92). Both the classic and alternative complement pathways are important in defense against *H. influenzae* type b. Protection against Hib appears to correlate with the concentration of anti-PRP antibody, with a serum antibody concentration of 0.15–1.0 μg/mL considered protective against invasive infection (93). Unimmunized infants and young children aged between 6 months and 5 years usually lack this level of anti-PRP antibody and are therefore susceptible to invasive Hib disease (94). Following immunization in infancy (<12 months of age), antibody levels fall by 18 months of age, below protective levels (95). After a booster dose in the 2nd year of life, however, high antibody levels persist for up to 8–10 years (95–97). Unlike the rise and subsequent decline in antibody levels seen after booster doses of vaccine, antibody avidity appears to increase more consistently over time following vaccination. After vaccination with Hib conjugate vaccine at age 2, 3, and 4 months of age, antibody avidity increases over time even while antibody levels fall (98). After a booster dose, both avidity and levels increased in this study, although lower avidity was evident in children with low antibody levels after initial priming doses, suggesting high avidity may be a marker of better priming. In addition, lower antibody avidity has been reported in vaccine failures (99), highlighting the importance of antibody function as well as concentration, although the level of antibody produced post-vaccination remains the sole basis for vaccine licensure.

### Non-type b *H. influenzae*

Much less is known about immunity to other *H. influenzae* serotypes or to non-typable isolates, which lack a polysaccharide capsule. Non-typable bacteria are very uncommon in invasive disease, and usually cause mucosal infections such as otitis media and sinusitis. For these strains, evidence suggests that antibodies directed against OMPs are bactericidal and protect against experimental challenge (100, 101). Disease caused by *H. influenzae* type a (Hia) has been most commonly reported in indigenous populations of North America, the reasons for which are unclear (102). In a recent study in Canada, SBA activity in Aboriginal adults was higher than in non-Aboriginal adults, suggesting higher rates of exposure and that exposure results in production of SBA (103). In this study, the SBA activity appeared to be mediated by IgM rather than IgG. This is akin to what has been described for *S. pneumoniae*, but contrasts with SBA against *N. meningitidis*, which is mediated by IgG. The precise role of this IgM-mediated SBA in protection against disease is not clear and further studies are awaited. Development of a protein-polysaccharide conjugate vaccine against Hia is ongoing, so determination of mechanisms of protection against Hia remains an important area of research (104, 105).

## Group B streptococcus

There is currently no licensed vaccine for protection against GBS and one of the reasons for this is the mechanism of protection is less definite then for the other encapsulated bacteria described. An added difficulty is that any studies of GBS immunity need to involve the mother-infant dyad.

An association between anti group B-polysaccharide antibody levels and invasive GBS disease in newborns was first described in 1976 (106). In most studies, low levels of anti-polysaccharide antibodies occurred in women who had neonates with GBS disease, compared with women with unaffected infants. There is a high correlation of antibody levels between the mothers and infants, indicating the importance of transplacental antibody transfer in neonatal immunity to GBS. In a recent meta-analysis the odds ratios of having an antibody level <2 μg/ml were 6.6 (95% CI: 2.1–20.6) and 2.4 (95% CI: 1.2–4.7) among those with types III and Ia GBS disease, respectively, compared to those without GBS disease (107). For capsule types Ia and III, one study suggested a threshold of 1 μg/ml as a correlate for protection (108), but much higher thresholds have been identified in other studies using different case-control designs (109, 110) and different methods, making direct comparisons difficult (111). Levels of polysaccharide-specific antibodies correlate with *in vitro* killing activity and *in vivo* protection (108), although several studies have suggested that some laboratory antibody detection methods may underestimate protection (112–114). In one study OP activity of anti-GBS polysaccharide IgG declined significantly from a 4-week post-immunization peak, but substantial functional activity (>1-log reduction in GBS cfu/mL), was preserved at 18–24 months post-immunization for each GBS type assessed (112). Animal challenge models (mostly in mouse, but also rat and rabbit) have reported that GBS killing is mediated by antibody- and complement-dependent OP via neutrophils.

Significant gaps remaining in understanding protection against invasive GBS disease. Interpretation of studies done to date is confounded by variation in methodologies and lack of standardized reference ranges for serotype-specific antibody levels. Prospective studies in diverse settings are needed to establish thresholds of protection for the most common serotypes IgG capsular antibodies are unlikely to be the only determinant of protection, so measures of functional antibodies are also required. Such work could facilitate the licensure pathway of a GBS vaccine without the need for large-scale efficacy trials in pregnant women (115).

## Concluding remarks

Antigen-specific antibody directed at the capsular polysaccharide clearly has the central role in protection against invasive infection by encapsulated bacteria, in both children and adults. Antibody-dependent mechanisms mediating this protection differ between organisms, with OP predominant in protection against *S. pneumoniae* and SBA against *N. meningitidis*. The precise mechanism is less-well defined for *H. influenzae* and GBS, and further information for these pathogens will aid development of vaccines against *H. influenzae* type a and GBS. Data from studies to date highlight that even in these cases no single mechanism is exclusively responsible for protection. The role of complement is also critical in disease protection, highlighted by the increased rates of severe infection against encapsulated bacteria in individuals with complement deficiencies. Protection against colonization and mucosal infections from these same pathogens is less well-understood and further studies are required, to inform development of vaccines which are better able to prevent these infection, for example pneumococcal pneumonia in older adults and vaccines based on non-polysaccharide antigens. Increased understanding of protection against non-b serotypes of *H. influenzae* and GBS would also be useful in development of new vaccines against these infections.

## Author contributions

MS is the sole author and was responsible for conceiving the idea for the manuscript and writing the manuscript.

### Conflict of interest statement

MS is supported via salary awards from the BC Children's Hospital Foundation, the Canadian Child Health Clinician Scientist Program and the Michael Smith Foundation for Health Research. MS has been an investigator on studies funded by Pfizer, Merck, VBI Vaccines, and GSK. All funds have been paid to his institute, and he has not received any personal payments.
